# Korean Red Pine (*Pinus densiflora*) Bark Extract Attenuates A&beta;-Induced Cognitive Impairment by Regulating Cholinergic Dysfunction and Neuroinflammation

**DOI:** 10.4014/jmb.2207.07015

**Published:** 2022-08-02

**Authors:** Min Ji Go, Jong Min Kim, Jin Yong Kang, Seon Kyeong Park, Chang Jun Lee, Min Ji Kim, Hyo Rim Lee, Tae Yoon Kim, Seung Gyum Joo, Dae-Ok Kim, Ho Jin Heo

**Affiliations:** 1 Division of Applied Life Science (BK21), Institute of Agriculture and Life Science, Gyeongsang National University, Jinju 52828, Republic of Korea; 2 Advanced Process Technology and Fermentation Research Group, World Institute of Kimchi, Gwangju 61755, Republic of Korea; 3 Korea Food Research Institute, Wanju-gun 55365, Republic of Korea; 4 Department of Food Science and Biotechnology, Kyung Hee University, Yongin 17104, Republic of Korea

**Keywords:** Korean red pine bark extract, amyloid beta, cognitive dysfunction, neuroprotective effect, cholinergic system, mitochondrial dysfunction

## Abstract

In this study, we investigated the anti-amnesic effect of Korean red pine (*Pinus densiflora*) bark extract (KRPBE) against amyloid beta_1-42_ (Aβ_1-42_)-induced neurotoxicity. We found that treatment with KRPBE improved the behavioral function in Aβ-induced mice, and also boosted the antioxidant system in mice by decreasing malondialdehyde (MDA) content, increasing superoxide dismutase (SOD) activities, and reducing glutathione (GSH) levels. In addition, KRPBE improved the cholinergic system by suppressing reduced acetylcholine (ACh) content while also activating acetylcholinesterase (AChE), regulating the expression of choline acetyltransferase (ChAT), postsynaptic density protein-95 (PSD-95), and synaptophysin. KRPBE also showed an ameliorating effect on cerebral mitochondrial deficit by regulating reactive oxygen species (ROS), mitochondrial membrane potential (MMP) and ATP levels. Moreover, KRPBE modulated the expression levels of neurotoxicity indicators Aβ and phosphorylated tau (p-tau) and inflammatory cytokines TNF-α, p-IκB-α, and IL-1β. Furthermore, we found that KRPBE improved the expression levels of neuronal apoptosis-related markers BAX and BCl-2 and increased the expression levels of BDNF and p-CREB. Therefore, this study suggests that KRPBE treatment has an anti-amnestic effect by modulating cholinergic system dysfunction and neuroinflammation in Aβ_1-42_-induced cognitive impairment in mice.

## Introduction

Alzheimer’s disease (AD), which is accompanied by problems in concentration, language, judgement, memory, and cognitive abilities, is the most common type of neurodegenerative disease [1]. There are reportedly approximately 50 million AD patients worldwide, and this is expected to increase to nearly 152 million people by 2050 [2]. Although the pathological cause of AD is still not clearly known, it is believed that various factors such as the formation and accumulation of amyloid beta (Aβ) and phosphorylated tau, synaptic dysfunction, mitochondrial damage, and inflammatory response complexly influence the development of AD [3]. Among Aβ peptides, Aβ_1-40/42_ are mainly produced via sequential decomposition of the amyloid precursor protein (APP) by β-secretase and γ-secretase [4]. The cleaved Aβ peptides aggregate into insoluble oligomers to form neuronal plaques, and present neurotoxicity, production of oxidative stress, synaptic plasticity disruption, tau hyperphosphorylation, and mitochondrial cytotoxicity. The continuous aggregation of these Aβ peptides induces memory loss and cognitive impairment, and ultimately leads to AD [5]. In addition, Aβ accelerates the formation of neurofibrillary tangles (NFTs) by activating tau kinase, leading to hyperphosphorylation of the tau associated with microtubule-related protein [6, 7]. Aβ accumulation induces the migration of microglia, which promotes the formation of reactive oxygen species (ROS) and pro-inflammatory cytokines such as tumor necrosis factor alpha (TNF-α) and interleukin-1β (IL-1β), and ultimately induces neuronal cell death [8]. Although there is currently no effective treatment for AD [9], the consumption of antioxidants is thought to prevent AD by suppressing its pathogenesis and progression [10].

Korean red pine (*Pinus densiflora*) bark has mostly been discarded as a byproduct of the forestry and timber industry [11]. However, Korean red pine bark extract (KRPBE) has various phenolic and flavonoids compounds with strong antioxidant activities [12]. The main physiologically active substances found in KRPBE include catechin, taxifolin, protocatechuic acid (PCA), vanillin, and procyanidin B1 [13, 14]. In particular, the phenolic compounds catechin, PCA, taxifolin and vanillin have exhibited high antioxidant activity and neuroprotective effects by inhibiting the activity of acetylcholinesterase (AChE) in vitro [13]. Although these various physiological activities, including the antioxidant, anti-inflammatory, anti-obesity, and immune-enhancement effects of KRPBE have been reported [13, 15, 16], there are few studies on the anti-amnesic effects of pine bark extract against Aβ-induced neurotoxicity. Therefore, this study was conducted to measure the anti-amnesic effect of KRPBE against Aβ-induced cognitive decline in Institute of Cancer Research (ICR) mice.

## Materials and Methods

### Chemicals

Aβ_1-42_, sodium chloride, potassium chloride, thiobarbituric acid (TBA), 2-[4-(2,4,4-trimethylpentan-2-yl)phenoxy]ethanol (Triton X-100), phenylmethane sulfonyl fluoride (pMSF), ethylenediaminetetraacetic acid (EDTA), metaphosphoric acid, 2-amino-2-(hydroxymethyl)propane-1,3-diol, o-phthaldialdehyde (OPT), hydroxylamine hydrochloride, sodium hydroxide, Iron (III) chloride hexahydrate, 5,5’dithiobis-(2-nitrobenzoic acid) (DTNB), 2-(acetylsulfanyl)-N,N,N-trimethylenthan-1-aminium (acetyl thiocholine), mannitol, sucrose, bovine serum albumin (BSA), HEPES sodium salt, 3,12-Bis(carboxymethyl)-6,9-dioxa-3,12-diazatetradecane-1,14-dioic acid (EGTA), digitonin, thichloroacetic acid (TCA), potassium dihydrogen phosphate, malate, HEPES, magnesium dichloride, pyruvate, 2’,7’-dicholorofluorescin diacetate, pyruvate, malate, tetraethylbenzimidazolyl-carbocyanine iodide (JC-1), protease inhibitor, polyvinylidene difluoride (PVDF) membrane, sodium azide and all other chemical products were purchased from Millipore (USA). Primary antibodies (acetylcholinesterase, sc-373901; postsynaptic density protein-95 (PSD-95), sc-32290; synaptophysin (SYP), sc-17750; β-amyloid, sc-374527; p-tau, sc-12952; phospho-protein kinase B (p-Akt)1/2/3, sc-514032; phosphor-c-Jun N-terminal kinase (p-JNK), sc-6254; phosphor-nuclear factor of kappa light polypeptide gene enhancer in B-cells inhibitor-alpha (p-IκB-α), sc-8404; cyclooxygenase-2 (COX-2), sc-376861; interleukin 1 beta, sc-515598; Bcl-2-associated X protein (BAX), sc-7480; B-cell lymphoma 2 (BCl-2), sc-509 and β-actin, sc-69879) were purchased from Santa Cruz Biotechnology (USA). Brain-derived neurotrophic factor (BDNF) (CSB-PA05775A0Rb) was purchased from Cusabio Biotech (China), and anti-phospho-cAMP response element-binding protein (p-CREB) (9198) and anti-rabbit (7074S) were purchased from Cell Signaling Technology (USA). Anti-choline acetyltransferase (ChAT)(20747-1AP) was purchased from Proteintech Group (USA), and anti-mouse (#1706516) was purchased from Bio-Rad (USA).

### Sample Preparation

Powdered KRPBE used in the experiment was obtained from Nutrapharm Co., Ltd. (Korea) on May 11, 2021. KRPBE was extracted by water, and stored at -20°C until use. In a previous study, procyanidin B1, catechin, taxifolin, protocatechuic acid, vanillin, and caffeic acid were identified as the major phenolic compounds in KRPBE [11].

### Animal Experimental Design and Protocols

**Animal Experimental Design**

ICR mice (male, 4 weeks) were obtained from Samtako Inc. (Korea). The mice were bred in a constant environment in which day and night were alternated at 12 h intervals at a temperature of 22 ± 2°C with relative humidity of 50-55%. There were five groups (ex vivo test, *n* = 5; mitochondrial experiment test, *n* = 3). The normal control (NC) and Aβ groups were orally administered with drinking water, and the normal sample (NS) was orally administered with KRPBE (30 mg/kg of BW dissolved in drinking water). The KRPBE groups were orally administered with amounts 15 (P15) and 30 (P30) mg/kg of BW dissolved in drinking water. All animal experiment processes were approved by the Animal Care and Use Committee of Gyeongsang National University (Certificate No. GNU-210616-M0059) and performed according to the provision of the Policy of the Ethical Committee of the Ministry of Health and Welfare, Korea. After the behavioral experiments, mice were sacrificed using CO_2_ inhalation for ex vivo tests.

### Aβ_1-42_-Induced AD Mice Model

Aβ_1-42_ dissolved in 0.89% NaCl solution was incubated at 37°C for 3 days for Aβ aggregation. Then, Aβ was injected to a depth of 2.5 mm in bregma with a Hamilton micro-syringe fitted with a 26-gauge needle (10 μl, intracerebroventricular (i.c.v.) injected). The NC and NS groups were injected with 10 μl of 0.89% NaCl without Aβ solution, and the Aβ, P15 and P30 groups were injected with 10 μl of 0.89% NaCl, including 410 pM Aβ solution [17]. A behavioral experiment was performed 3 days after Aβ injection to allow the experimental animals to recover from surgical treatment.

### Y-Maze Test

The Y-maze test was conducted using a maze device with three arms (length = 33 cm; height = 15 cm; width = 10 cm) made of black plastic material, and each arm was randomly designated as either A, B, or C zone. The mice were carefully placed on one arm and the movement path into each arm was recorded using a video tracking system (Smart v3.0, Panlab, Spain) for 8 min [18].

### Passive Avoidance Test

To perform the passive avoidance test, a device consisting of two zones with a light and a dark chamber was used. The bottom of the device was made of stainless steel to produce an electric shock. During the training trial, mice were acclimatized to the dark environment and device for 1 min. Then, the light was turned on and the mice were exposed to light for 2 min, and the door between the two chambers was opened [19]. An electric shock of 1.5 mA was applied for 3 s after the mice had moved completely, including all four feet, from the light chamber to the dark chamber [20]. After 24 h, in the test trial, each mouse was placed in the light chamber in the same way as in the training trial, and the latency time for all four paws of the mice to enter the dark chamber was measured for up to 300 s [21].

### Morris Water Maze Test

The Morris water maze test consisted of a circular water tank (diameter = 150 cm; height = 60 cm) divided into four quadrants and filled with 30 cm of opaque water containing dissolved squid ink (Cebesa, Spain) (23 ± 2°C). Visual clues were displayed along the divided quadrants, and a black escape platform was placed in the center of the W zone. In the visible trial, the platform was exposed 1 cm above the surface of the water, and each mouse swam for up to 60 s until finding it. After that, a training trial was conducted for 4 days with the platform set to be invisible 1 cm below the surface of the water, and training was repeated four times a day. In the probe trial, the time the mice stayed in the W zone was recorded for 60 s without the platform [22]. The movements of mice were recorded using a video tracking system (Smart v3.0, Panlab).

### Preparation of Tissue

After the behavioral test, mice brain tissue was immediately isolated for biochemical analysis. The brain tissue was homogenized using a bullet blender (Next Advance Inc., USA) with 10X volume of phosphate-buffered saline (PBS, pH 7.4). The protein concentration of the obtained sample was calculated according to the Bradford method [23].

### Measurement of Antioxidant System Biomarker

For the measurement of superoxide dismutase (SOD) activity, the brain tissue was centrifuged at 400 ×*g* for 10 min at 4°C, and the obtained pellet was reacted to 1X cell extraction buffer with 20% (v/v) Triton X-100, distilled water, and 200 mM PMSF. The mixture was vortexed for 30 min on ice. After centrifugation at 10,000 ×*g* for 10 min at 4°C, the supernatant was obtained. Then, the SOD content was measured using an SOD kit (Sigma-Aldrich Chemical Co., USA).

To measure the reduced glutathione (GSH) content, the brain tissue homogenate was mixed with 200 μM phosphate buffer (pH 6-7) and centrifuged at 10,000 ×*g* for 15 min at 4°C. The protein concentration of the supernatant was quantified. Then, the supernatant and 5% metaphosphoric acid were mixed at a 1:1 ratio, and the mixture was centrifuged at 2,000 ×*g* for 2 min at 4°C. The reduced GSH was measured using the obtained supernatant. The supernatant was reacted with 0. 26 M Tris-HCl (pH 7.5), 0.65 N NaOH and 1 mg/ml OPT in methanol for 15 min. The fluorescence of reactants was measured at an excitation wavelength of 360 nm and an emission wavelength of 430 nm using a fluorescence photometer (Infinite F200, Tecan, Switzerland) [24].

For the malondialdehyde (MDA) level measurement, the brain tissue homogenate was mixed with PBS buffer and centrifuged at 2,356 ×*g* for 10 min at 4°C, and the supernatant was used for the experiment. The supernatant was mixed with 1% phosphoric acid and 0.67% TBA, and it was bathed at 95°C for 1 h. Then, the MDA content of the mixture was measured at 532 nm using a microplate reader (Epoch2, BioTek, USA) [25].

### Measurement of ACh Content and AChE Inhibitory Activity

The brain tissue homogenate was centrifuged at 13,572 ×*g* for 30 min at 4°C, and the supernatant was reacted with alkaline hydroxylamine reagent (2 M hydroxylamine in 1 N HCl and 3.5 N sodium hydroxide, 1:1 mixture). Then, the mixture was mixed with 0.5 N HCl and 0.37 M FeCl_3_ · 6H_2_O in 0.1 N HCl, and the acetylcholine (ACh) content was measured at 540 nm using a microplate reader (Epoch2, BioTek) [26].

To measure the acetylcholinesterase (AChE) inhibitory activity, the brain tissue homogenate was added to 50 mM sodium phosphate buffer and incubated at 37°C for 15 min. After incubation, the incubated mixture was reacted with Ellman’s reaction mixture (500 μM acetylthiocholine with 1 mM DTNB at 37°C for 10 min, and the reaction solution was measured at 405 nm for 20 min using a microplate reader (Epoch2, BioTek) [27].

### Extraction of Mitochondrial from Brain Tissue

For mitochondrial extraction, brain tissue was homogenized using a bullet blender (Next Advance Inc.) by mixing mitochondrial isolation (MI) buffer that included 215 mM mannitol, 75 mM sucrose, 0.1% BSA and 20 mM HEPES sodium salt (pH 7.2) with 1 mM EGTA, which was then centrifuged at 1,300 ×*g* for 5 min at 4°C to obtain the supernatant. The mixture was centrifuged again at 13,000 ×*g* for 10 min at 4°C, and the pellet was mixed with 0.1% digitonin and MI buffer containing 1 mM EGTA and reacted for 5 min. The mixture was centrifuged at 13,000 ×*g*, for 15 min at 4°C. The pellet was mixed with MI buffer, and mitochondrial function was evaluated using the mixture [28].

### Assessment of Mitochondrial Activity

To investigate the reactive ROS level in mitochondria, mitochondrial extract was reacted with a respiration buffer including 125 mM KCl, 2 mM KH_2_SO_4_, 2.5 mM malate, 20 mM HEPES, 1 mM MgCl_2_, 5 mM pyruvate, 500 μM EGTA, and 25 μM DCF-DA. Then, the reactant was incubated in a dark room for 20 min, and fluorescence was measured using a fluorometer (Infinite F200, Tecan) at 485 nm (excitation wavelength) and 535 nm (emission wavelength) [29].

To measure the mitochondrial MMP level, the mitochondrial extract was reacted with MI buffer with 5 mM pyruvate, 5 mM malate, and 1 μM JC-1, and gently shaken. Then, the reactant was incubated in a dark room for 20 min, and fluorescence was measured at 530 nm (excitation wavelength) and 590 nm (emission wavelength) using a fluorometer (Infinite F200, Tecan) [30].

The ATP level was measured using an ATP kit (Sigma-Aldrich Chemical Co.) according to the manufacturer’s protocol. The reactant was then measured with a luminescence meter (GloMax, Promega, USA).

### Western Blot Assay

Whole brain tissue was homogenized by mixing with ProtinEx Animal Cell/Tissue (Gene All Biotechnology, Korea) containing 1% protease inhibitor followed by centrifugation at 13,000 ×*g* for 10 min at 4°C. Then, the sample was separated by sodium dodecyl sulfate-polyacrylamide gel electrophoresis (SDS-PAGE), and the separated protein was transferred to a PVDF membrane (Millipore). To block the membrane, it was treated with 5% skim milk for 1 h, and then washed three times for 10 min using 1X Tris-buffered saline with 0.1% Tween® 20 (TBST) buffer. The membrane and primary antibody (1:1,000) solution were incubated for 12 h at 4°C. After incubation, secondary antibody (1:2,500) was reacted with the membrane for 1 h at room temperature, and the chemiluminescence band was detected using an image analyzer (iBright CL1000, Invitrogen, USA). Finally, the density of the band was calculated using ImageJ software (National Institutes of Health, USA).

### Statistical Analysis

All data were expressed as mean ± SD and were calculated and displayed by one-way analysis of variance (ANOVA) to analyze significant differences between groups. Significant differences were confirmed in each data set using Duncan’s new multiple range test (*p* < 0.05) with the SAS program (version 9.4, SAS Institute Inc., USA), and different small letters mean statistically significant differences.

## Results

### Y-Maze Test

**Fig. 1:**
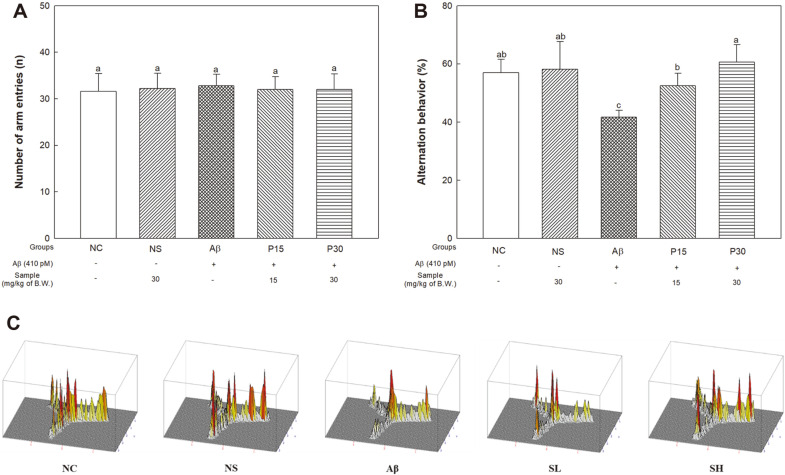
Protective effect of KRPBE on Y-maze in Aβ_1-42_-induced mice. (**A**) Alternation behavior, (**B**) Number of arm entries, and (**C**) 3D moving routes. (**C**) 3D moving routes. The results shown are mean ± SD (*n* = 5). Data were statistically considered at *p* < 0.05, and different small letters represent statistical difference.

The Y-maze test was conducted to assess the protective effect of KRPBE against Aβ-induced impairment of spatial working ability (Fig. 1). Since there was no significant difference between groups in the total number of times the arms were entered, we confirmed that exercise ability did not affect the experimental results (Fig. 1A). As a result of alternative behavior, there was no significant difference between the NC (56.97%) and the NS groups (58.13%) (Fig. 1B). On the other hand, the Aβ group (41.66%) significantly decreased compared to the NC group. The P15 and P30 groups (52.49% and 60.67%, respectively) significantly increased compared to the Aβ group. Also, we observed that the Aβ group was biased toward one arm compared to the other groups in the 3D route-tracking picture (Fig. 1C).

### Passive Avoidance Test

**Fig. 2:**
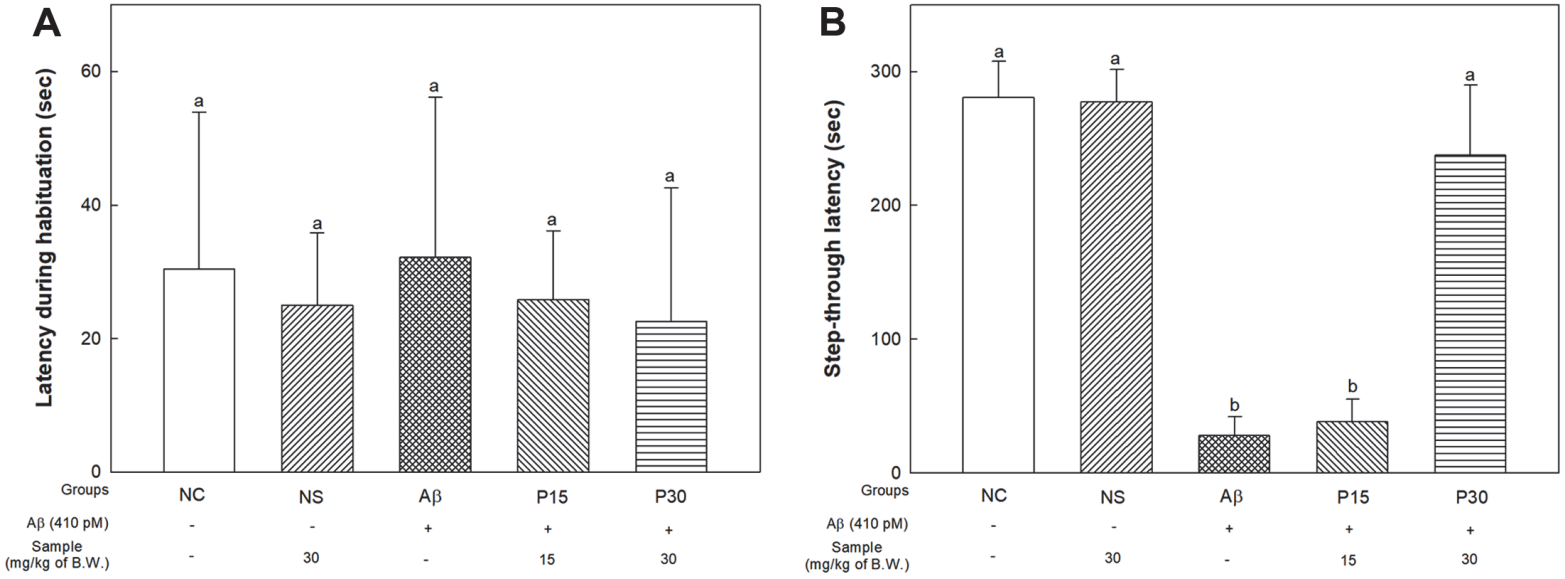
Protective effect of KRPBE on passive avoidance test in Aβ_1-42_-induced mice. (**A**) Latency during habituation and (**B**) Step-through latency. The results shown are mean ± SD (*n* = 5). Data were statistically considered at *p* < 0.05, and different small letters represent statistical difference.

The passive avoidance test was performed to measure the improvement effect of KRPBE on Aβ-induced short-term memory impairment (Fig. 2). There was no significant difference between groups in the latency time entering the dark chamber during the training session (Fig. 2A). As a result of measuring step-through latency in the test trial, there was no significant difference between the NC (280.80 s) and NS groups (277.20 s) (Fig. 2B). The Aβ group (28.00 s) significantly decreased compared to the NC group (280.80 s). On the other hand, the P30 group (237.80 s) showed significantly improved short-term memory compared to the Aβ group and P15 group (38.40 s).

### Morris Water Maze Test

**Fig. 3:**
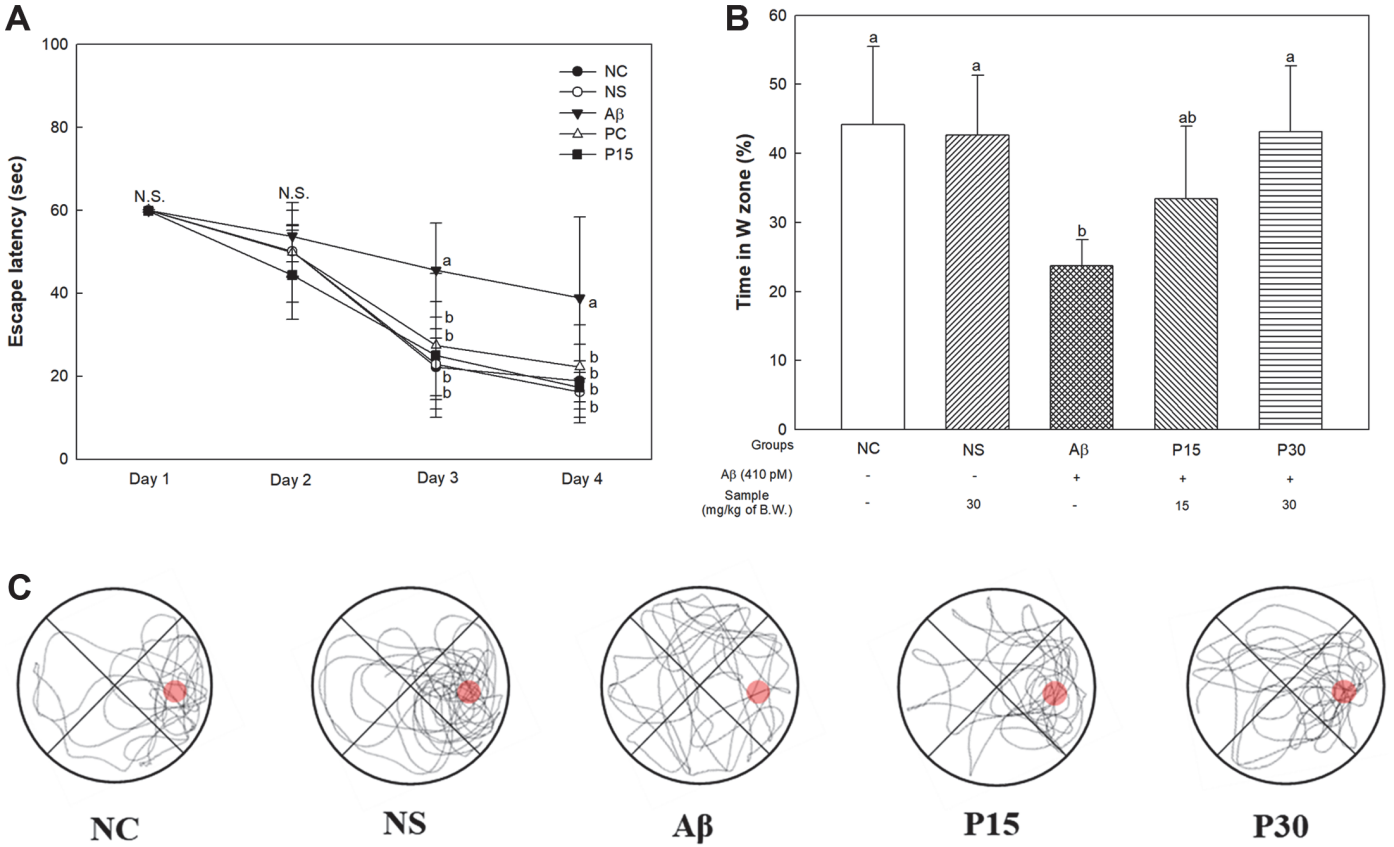
Protective effect of KRPBE on Morris water maze test in Aβ_1-42_-induced mice. (**A**) Escape latency in the training session, (**B**) Retention time on W zone in the prove session, and (**C**) Path tracking of each group in the probe trail. The results shown are mean ± SD (*n* = 5). Data were statistically considered at *p* < 0.05, and different small letters represent statistical difference.

The Morris water maze test was performed to investigate long-term memory ability (Fig. 3). In the training trial, we confirmed that the escape latency time decreased as the training was repeated for all groups, and the Aβ group (38.88 s) had the most delayed escape latency time compared to other groups (Fig. 3A). The time spent in the W zone in the probe session did not show a significant difference between the NC group (44.19 s) and the NS group (42.64 s), but it was confirmed that the Aβ group (23.75 s) had a decreased time compared to these groups (Fig. 3B). However, the P15 group (33.47 s) showed partial increase, and the P30 group (43.15 s) had significantly increased retention time compared to the Aβ group. Similar to the previous results, the Aβ group showed a tendency to move in a variety of quadrants in the 3D path tracking, while the other groups insistently stayed in the W zone (Fig. 3C).

### Antioxidant System Biomarkers

**Fig. 4:**
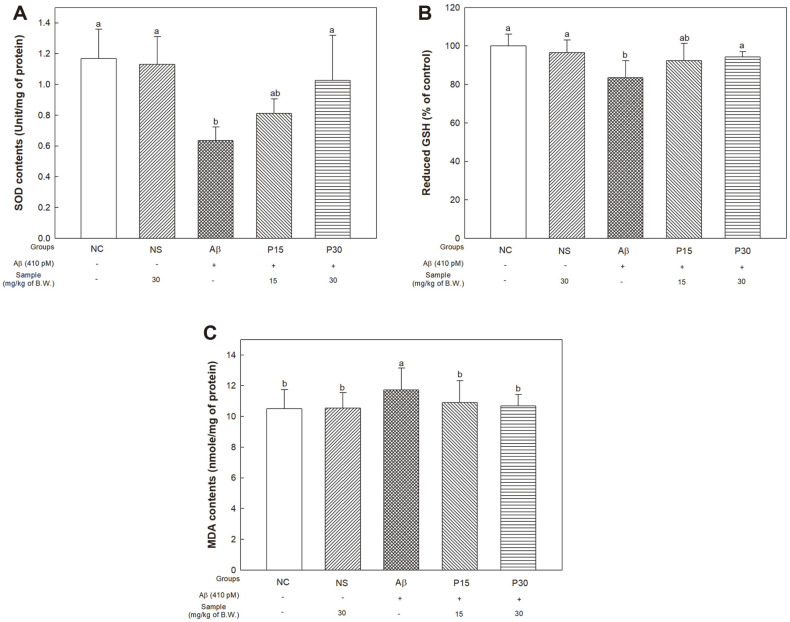
Protective effect of KRPBE on Aβ_1-42_-induced biomarkers changes related with antioxidant system. (**A**) SOD contents, (**B**) Reduced GSH level, and (**C**) MDA contents. The results shown are mean ± SD (*n* = 5). Data were statistically considered at *p* < 0.05, and different small letters represent statistical difference.

To measure the improvement effect of KRPBE on antioxidant system biomarkers, we evaluated the SOD content and reduction of GSH and MDA levels (Fig. 4).

The SOD content results showed no significant difference between the NC group (1.17 unit/mg of protein) and the NS group (1.13 unit/mg of protein) (Fig. 4A). We confirmed that the Aβ group (0.64 unit/mg of protein) significantly decreased compared to these groups. On the other hand, the P30 group (1.03 unit/mg of protein) showed increased SOD content compared to that of the Aβ group.

The results for reduction of GSH level showed no significant differences between the NC (100% of control) and NS groups (96.60% of control) (Fig. 4B). However, the Aβ group (83.47% of control) revealed a significant decrease compared to the NC group, while the P15 group (92.33% of the control) showed a partial increase, and the P30 group (94.30% of control) showed a significant increase in comparison with the Aβ group.

In the results for MDA levels, there was no significant difference between the NC group (10.50 nmole/mg of protein) and NS group (10.55 nmole/mg of protein) (Fig. 4C). The Aβ group (11.73 nmole/mg of protein) showed an increase compared to the NC group, whereas the P15 and P30 groups (10.90 nmole/mg of protein and 10.67 nmole/mg of protein, respectively) demonstrated improvement compared to the Aβ group.

### Cholinergic System and Synaptic Function Protective Effect

**Fig. 5:**
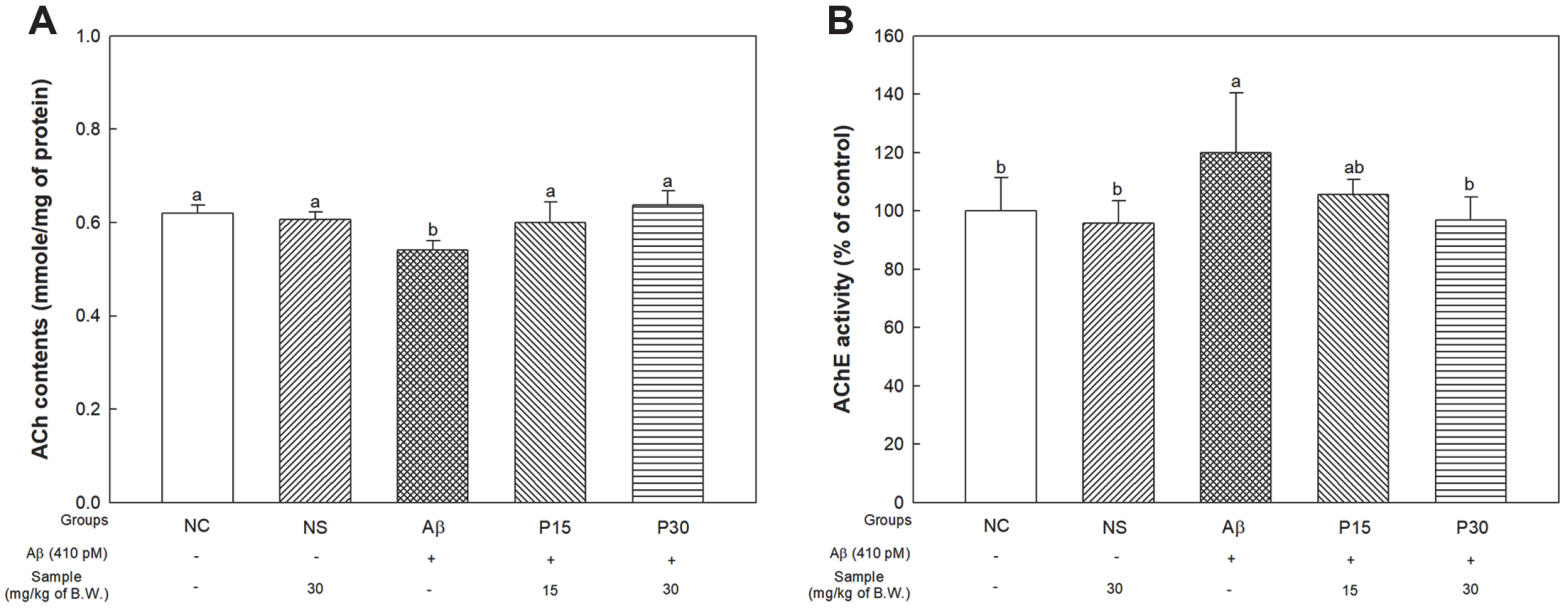
Protective effect of KRPBE on Aβ_1-42_-induced cholinergic dysfunction. (**A**) ACh content and (**B**) AChE activity. The results shown are mean ± SD (*n* = 5). Data were statistically considered at *p* < 0.05, and different small letters represent statistical difference.

To investigate the protective effect of KRPBE against cholinergic system dysfunction in Aβ-induced AD mice, we next evaluated ACh content and AChE activity (Fig. 5). There was no significant difference in ACh content between the NC group (0.62 nmole/mg of protein) and NS group (0.61 nmole/mg of protein), and the Aβ group (0.54 nmole/mg of protein) significantly decreased compared to the NC group (0.62 nmole/mg of protein) (Fig. 5A). On the other hand, the P15 and P30 groups (0.60 nmole/mg of protein and 0.64 nmole/mg of protein) improved compared to the Aβ group.

In determining AChE activity, we found no significant between the NC group (100%) and NS group (95.80%)(Fig. 5B). On the other hand, the Aβ group (120.04%) showed significant increase compared to the NC group. Additionally, the P15 group (105.63%) showed partial decrease compared to the Aβ group, while the P 30 group (96.73%) exhibited a significant decrease of AChE activity.

**Fig. 6:**
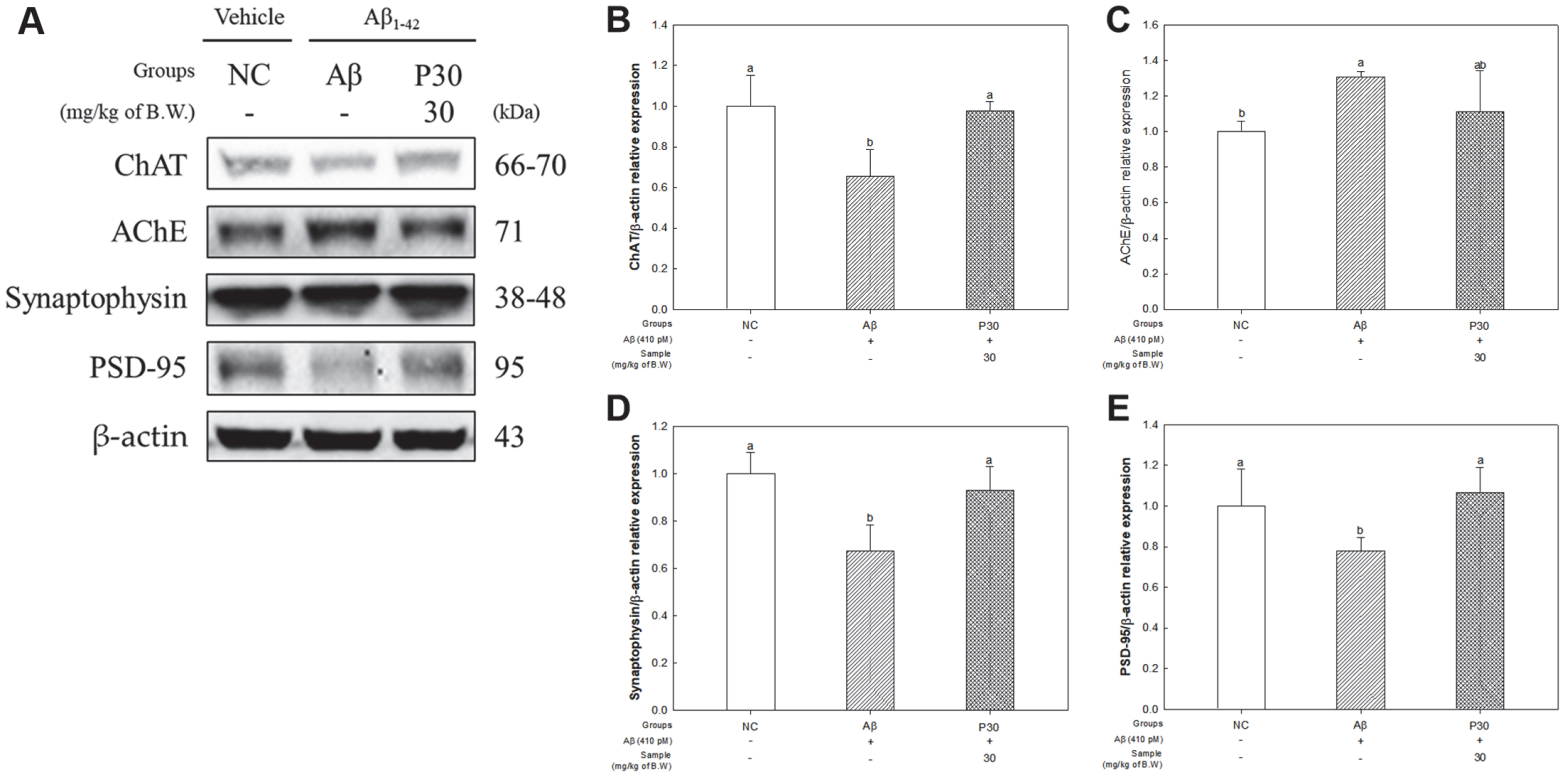
Effect of KRPBE on cholinergic system and synaptotoxicity related protein expression in western blot. (**A**) Western blot band image, Protein expression levels of (**B**) ChAT, (**C**) AChE, (**D**) Synaptophysin, and (**E**) PSD-95. The results shown are mean ± SD (*n* = 3). Data were statistically considered at *p* < 0.05, and different small letters represent statistical difference.

To evaluate the protective effect of KRPBE against Aβ toxicity-induced cholinergic system dysfunction and synaptotoxicity, we investigated the expression levels of ChAT, AChE, synaptophysin, and PSD-95 proteins (Fig. 6). The expression levels of ChAT (65.53%), synaptophysin (67.34%), and PSD-95 (77.82%) were significantly decreased in the Aβ group compared to the NC group, but expression levels in the P30 group (97.60%, 93.06% and 106.67%, respectively) were upregulated (Figs. 6B, 6D, and 6E). Also, in the results of AChE protein expression, the expression level of the Aβ group (130.56%) significantly increased compared to the NC group, and the expression level of the P30 group (111.13%) was partially downregulated compared to the Aβ group (Fig. 6C).

### Mitochondrial Activity

**Fig. 7:**
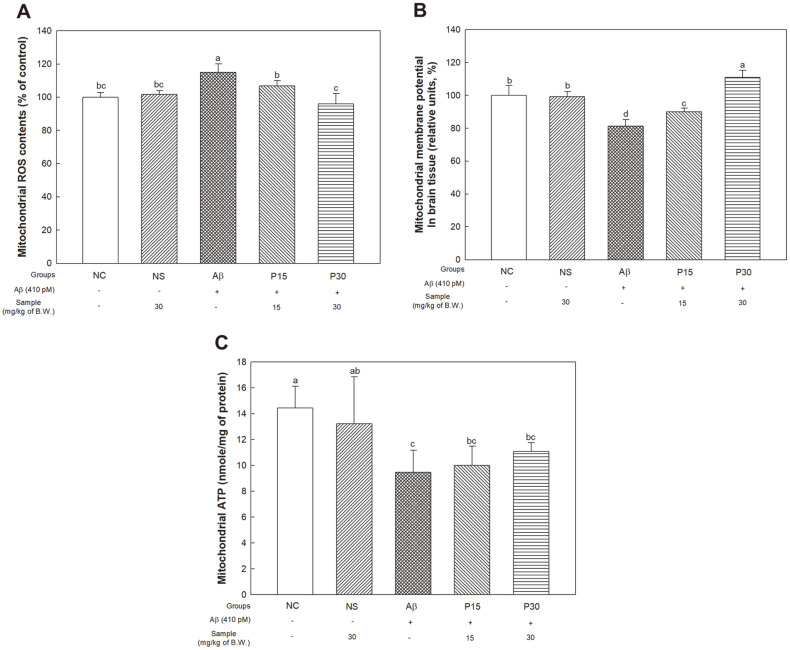
Protective effect of KRPBE on Aβ_1-42_-induced mitochondrial dysfunction. (**A**) ROS levels, (**B**) MMP activity, and (**C**) Mitochondrial ATP contents. The results shown are mean ± SD (*n* = 3). Data were statistically considered at *p* < 0.05, and different small letters represent statistical difference.

To evaluate the ameliorating effect of KRPBE on Aβ-induced mitochondrial dysfunction, we next investigated ROS, MMP and ATP levels (Fig. 7). In the mitochondrial ROS level in brain tissue, there was no significant difference between the NC group (100% of control) and the NS group (101.63% of control). We found significant increase in the Aβ group (114.98% of control) compared to the NC group (Fig. 7A). On the other hand, the P15 and P30 groups (106.84% of control and 96.00% of control, respectively) showed significant decrease compared to the Aβ group.

In the results for MMP level, there was no significant difference between the NC group (100%) and the NS group (99.30%). The Aβ group (81.28%) showed a decreased MMP level compared to the NC group (Fig. 7B). However, the P15 and P30 groups (90.03% and 110.01%, respectively) showed significant improvement in reduced MMP levels.

In the results for mitochondrial ATP content, there was no significant difference between the NC group (14.45 nmole/mg of protein) and the NS group (13.23 nmole/mg of protein). The Aβ group however (9.47 nmole/mg of protein) showed significant decrease compared to the NC group (Fig. 7C). The P15 group (10.00 nmole/mg of protein) and P30 group (11.07 nmole/mg of protein) exhibited increased ATP content, but there was no significant difference.

### Aβ Toxicity-Induced Neuroinflammatory-Related Factor

**Fig. 8:**
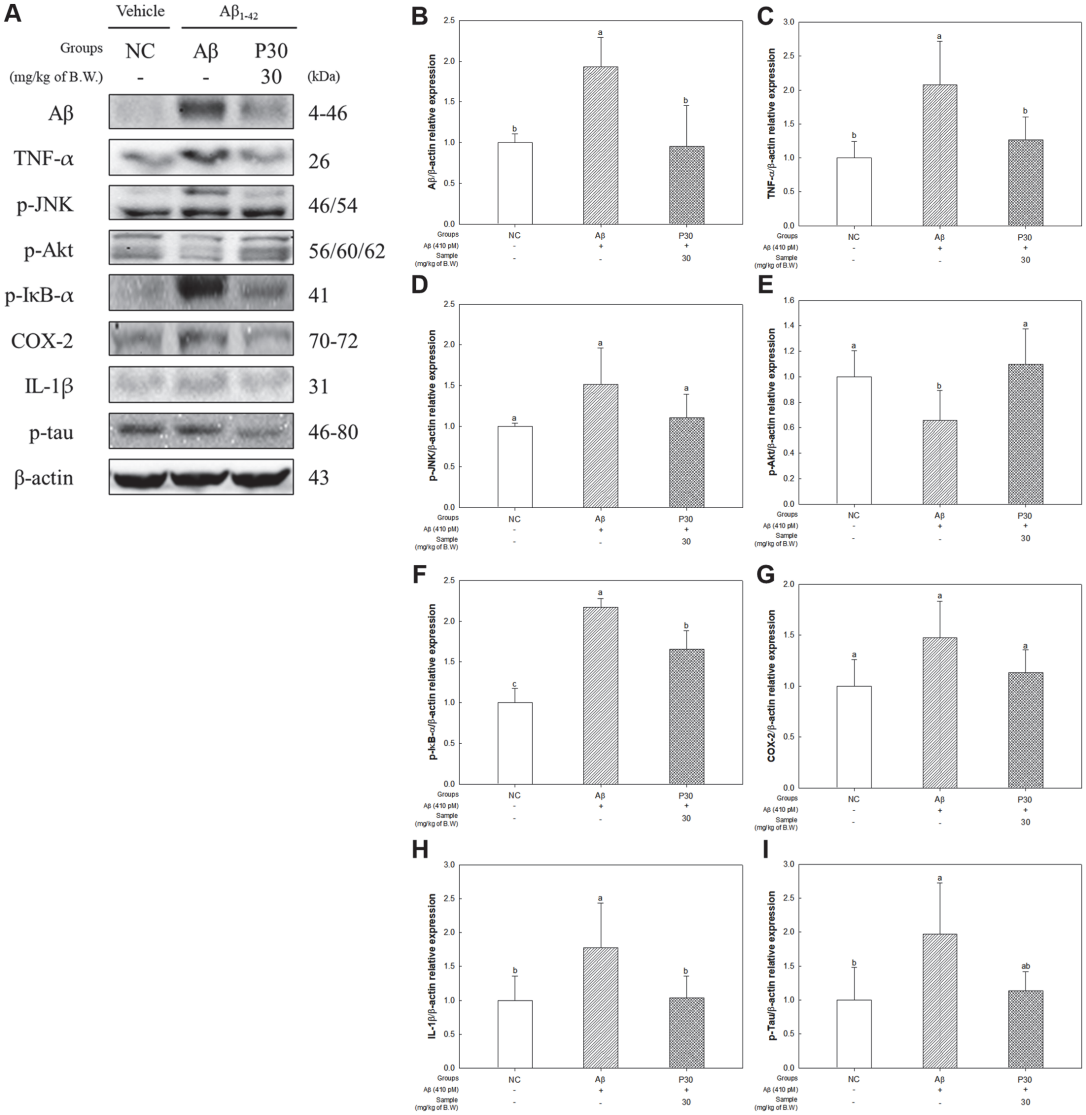
Effect of KRPBE on Aβ toxicity-induced neuroinflammatory related protein expression in western blot. (**A**) Western blot band image, Protein expression levels of (**B**) Aβ, (**C**) TNF-α, (**D**) p-JNK, (**E**) p-Akt, (**F**) p-IκB-α, (**G**) COX-2, (**H**) IL-1β, and (**I**) p-tau. The results shown are mean ± SD (*n* = 3). Data were statistically considered at *p* < 0.05, and different small letters represent statistical difference.

To investigate the protective effect of KRPBE against the Aβ toxicity-induced neuroinflammatory path, we next measured the protein expression levels of β-amyloid, TNF-α, p-JNK, p-Akt, p-IκB-α, COX-2, IL-1β, and p-tau (Fig. 8). β-amyloid and TNF-α protein expression levels were significantly increased in the Aβ group (193.27%and 207.63%, respectively) compared to the NC group (Figs. 8B and 8C). On the other hand, the expression level of β-amyloid and TNF-α were significantly deceased in the P30 group (95.29% and 126.72%, respectively) compared to the Aβ group. The expression levels of p-JNK in the Aβ group (151.44%) were increased compared to the NC group, and the P30 group (110.65%) showed a reduced expression level compared to the Aβ group (Fig. 8D). However, there was no significant difference between all groups. The expression level of p-Akt significantly decreased in the Aβ group (65.64%) compared to the NC group (Fig. 8E). However, the expression level in the P30 group (109.94%) significantly increased compared to the Aβ group. The expression levels of p-IκB-α (217.05%) and IL-1β (177.29%) in the Aβ group were significantly increased compared to the NC group (Figs. 8F and 8H), whereas the P30 group (16.62% and 103.86%, respectively) had significantly downregulated expression levels compared to the Aβ group. The expression levels of COX-2 in the Aβ group (147.77%) were increased compared to the NC group, and the P30 group (113.05%) showed a reduced expression level compared to the Aβ group (Fig. 8G). However, there was no significant difference between all groups. The p-tau protein expression level significantly increased in the Aβ group (197.22%) compared to the NC group (Fig. 8I), but the expression level of the P30 group (113.31%) was partially downregulated compared to the Aβ group.

### Apoptosis-Related Factors

**Fig. 9:**
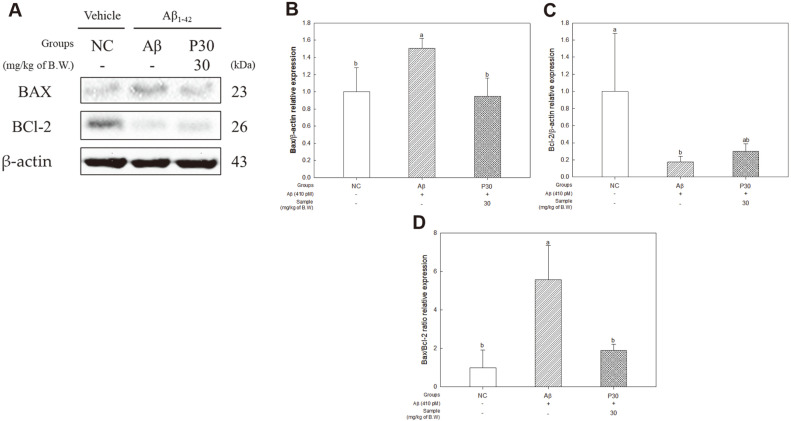
Effect of KRPBE on apoptosis-related protein expression in western blot. (**A**) Western blot band image, Protein expression levels of (**B**) BAX, (**C**) BCl-2, and (**D**) BAX/BCl-2 ratio. The results shown are mean ± SD (*n* = 3). Data were statistically considered at *p* < 0.05, and different small letters represent statistical difference.

To determine the protective effect of KRPBE against apoptosis induced by Aβ accumulation, we investigated the expression levels of BAX and BCl-2, and the BAX/BCl-2 ratio (Fig. 9). The expression levels of BAX (150.62%) in the Aβ group were significantly increased compared to the NC group (Fig. 9B). On the other hand, the expression levels of BCl-2 (17.36%) in the Aβ group were significantly decreased compared to those of the NC group, and the expression levels in the P30 group (29.91%) were significantly increased compared to those in the Aβ group (Fig. 9C). The expression levels of BAX/BCl-2 ratio (557.06%) in the Aβ group were significantly increased compared to those of the NC group, and the expression levels in the P30 group (189.67%) were significantly downregulated (Fig. 9D).

### BDNF/CREB Pathway-Related Factors

**Fig. 10:**
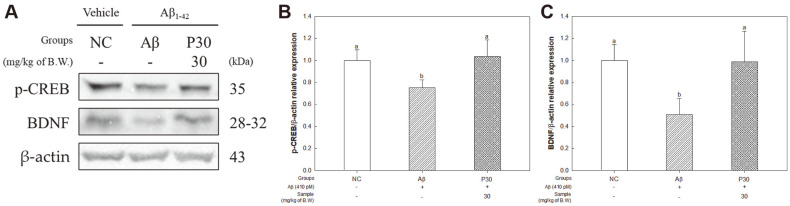
Protective effect of KRPBE on CREB/BDNF pathway-related protein expression in western blot. (**A**) Western blot band image, Protein expression levels of (**B**) p-CREB, and (**C**) BDNF. The results shown are mean ± SD (*n* = 3). Data were statistically considered at *p* < 0.05, and different small letters represent statistical difference.

To determine the regulatory effect of KRPBE via the BDNF/CREB pathway, we investigated the p-CREB and BDNF expression levels (Fig. 10). The expression levels of p-CREB (75.01%) and BDNF (50.76%) in the Aβ group were significantly reduced compared to those in the NC group (Figs. 10B and 10C). On the other hand, the P30 group (103.54% and 98.69%, respectively) showed a restored BDNF/CREB pathway through upregulation of the protein expression level.

## Discussion

The most common type of dementia, AD, mainly affects the elderly population [1, 31]. This disease is characterized by impairment of cognitive functions, such as learning and memory, and neurodegenerative dysfunction, including intellectual and emotional dysfunction [32]. Accumulation of Aβ in AD accelerates pathological changes, including the production of neurofibrillary tangles, synaptic degeneration, and impaired neuronal function in brain tissues [33, 34]. In addition, the accumulation of Aβ causes excessive oxidative stress, resulting in neuronal cell apoptosis and death [35]. Ultimately, neuronal cell death exacerbates the decline in memory and cognitive function in AD [36]. Therefore, in this study we investigated whether the administration of KRPBE could improve cognitive dysfunction as well as the antioxidant, mitochondrial, and cholinergic system disorders induced by Aβ toxicity.

Aβ induces oxidative stress and accumulates at synaptic terminals, leading to synaptic dysfunction [36, 37]. It also enters the synaptic mitochondria and activates the apoptotic pathway, leading to neuronal cell death and neurotransmission disorders [37]. In particular, it damages the hippocampus and cortical regions, which play a crucial role in spatial, working, and long-term memory, resulting in cognitive impairment [38, 39]. Therefore, in vivo behavioral tests were performed to evaluate the protective effect of KRPBE against Aβ-induced cognitive impairment. In this study, treatment with KRPBE in Aβ-induced cognitive decline mice showed significant cognitive improvements (Figs. 1-3). Similarly, *Pinus halepensis* essential oil improved Aβ-induced decline in working memory by improving the antioxidant and cholinergic system in rats [40]. Supplementation with *Pinus roxburghii* stem bark extract improved behavioral, cognitive, memory, biochemical and AChE expression in streptozotocin (STZ)-induced rats [41]. Treatment with French maritime pine (*Pinus maritima*) bark (pycnogenol, PYC), improved learning and memory ability by regulating intracerebroventricularly (ICV)-STZ-induced oxidative stress and cholinergic neurotransmission deficiency in the cerebral cortex and hippocampus of rats [42]. Furthermore, it was reported that procyanidin treatment helps relieve AD by improving cognitive function by regulating CREB-silent information regulator 1 (SIRT1) [43]. In particular, treatment with catechin hydrate, one of the major physiologically active substances of KRPBE, helped to prevent memory loss by upregulating antioxidant capacity and the expression of ChAT against ICV-STZ-induced neuronal loss and memory impairment [44]. Therefore, KRPBE, which contains various phenolic compounds and procyanidins, may have protective effects in Aβ-induced cognitive decline mice.

Oxidative stress, considered to be causative factor of AD, results in excessive production of ROS, DNA oxidation, and inhibition of antioxidant enzymes, such as SOD and glutathione peroxidase (GPx) [45]. Furthermore, mitochondrial oxidative stress is detected early in the progression of AD, before the onset of Aβ pathology [46]. A compromised antioxidant system loses its ability to inhibit lipid peroxidation in brain cell membranes, which affects AD neurodegeneration [47]. In particular, the brain consumes a large amount of oxygen and is very vulnerable to oxidative stresses such as iron, ascorbate and polyunsaturated fatty acids that produce free radicals [48]. Therefore, in this study, the SOD, reduced GSH and MDA levels, which are representative oxidative stress biomarkers, were evaluated to measure the protective effect of KRPBE against Aβ-induced oxidative stress (Fig. 4). Administration of KRPBE significantly regulates the activity of antioxidant systems by increasing SOD and GSH levels and reducing MDA levels. Similar to these results, *P. densiflora* bark extract improved the levels of GSH, the activity of SOD, GPx, and catalase (CAT) in selenite-induced rat pups [49]. Taxifolin found in French marine pine bark has also been reported to show significant scavenging activity of radicals and inhibition of lipid peroxidation in serum and liver [11, 50]. In particular, vanillin, a kind of bioactive compound in KRPBE, significantly increased GSH, SOD, CAT and GPx levels in rats with carbon tetrachloride-induced acute brain injury, as well as decreasing MDA levels, resulting in significant brain protection [51]. It is suggested that KRPBE can reduce Aβ-induced oxidative stress with the antioxidant system protective effect of pine bark extract.

Aβ-induced cholinergic system dysfunction in basal forebrain cholinergic neurons is a pathological characteristic of early AD [52]. The cholinergic system is implicated in several neural functions, such as learning, memory, and sleep, and ACh plays a role in regulating these functions [53]. However, Aβ peptides reduce the production and release of the neurotransmitter ACh and increase AChE activity, decomposing ACh to acetate and choline [54, 55]. AChE forms a stable complex with the Aβ enzyme (Aβ-AChE complex), and the Aβ-AChE complex promotes the degradation of ACh [39]. In addition, by increasing calcium in hippocampal neurons, it causes a loss of MMP and activates the apoptotic pathway [56]. Therefore, in this study, the ACh level and AChE activity were investigated to confirm the protective effect against Aβ-induced cholinergic system dysfunction (Fig. 5). KRPBE administration increased the ACh level and decreased AChE activity compared to the Aβ group. Similar to these results, for cholinergic dysfunction-induced Aβ administration, *P. halepensis* essential oil inhibited the activity of AChE [40]. In a previous study, Korean red pine bark extract, which contains taxifolin, vanillin, and catechin, inhibited the in vitro activity of AChE in a concentration-dependent manner. In particular, it was reported that catechin and vanillin significantly inhibited AChE activity [13]. Synaptophysin regulates activity-dependent synapse formation and is a synaptic vesicle membrane protein present throughout the brain. PSD-95 is a post-synaptic scaffolding protein that adjusts the development of synapse and neural plasticity, and plays a crucial role in the learning process [34, 57]. In addition, KRPBE ameliorated Aβ-induced synaptic dysfunction by regulating synaptophysin and PSD-95 via synaptic mechanisms (Fig. 6). Similar to these results, doses of pine stem bark extract significantly reduced the activity of AChE and improved ACh levels in STZ-induced Alzheimer's-type neurodegeneration and cognitive deficits [41]. In addition, the treatment of PYC showed a neuroprotective effect by reducing oxidative stress and preserving presynaptic proteins in both the cortex and hippocampus in a traumatic brain injury animal model [58]. Protocatechuic acid (PCA), a main physiologically active substance of KRPBE, protected diabetes-induced neurotoxicity and behavioral disorders by inhibiting the activity of AChE and inflammatory response in cerebral and cerebellar tissues in a diabetic rat model [59]. In addition, treatment with taxifolin, a bioactive phenolic compound of pine bark extract, improved synaptic dysfunction and cognitive impairment by reducing the expression of PSD-95 in Aβ42-induced AD model mice [60]. Based on these results, KRPBE due to its neuroprotective effects and ability to protect the cholinergic system and synaptic protein, can ultimately improve cognitive dysfunction.

Mitochondria, which are ATP-producing organelles, act in many cellular functions, such as redox reactions, free-radical scavenging of cells, and activation of the caspase-mediated cell death pathway [61]. Aβ accumulated in mitochondria and other organelles triggers the production of ROS and oxidative stress, leading to cellular dysfunction [37]. In particular, Aβ induces an inappropriate mitochondrial complex function through interaction with mitochondrial matrix components, and damages the MMP of organelles [46]. In addition, the decay of mitochondria by aging and oxidative stress can eventually impair ATP synthesis and cause the loss of mitochondrial cellular calcium homeostasis [62]. Therefore, this study was performed to assess the improvement effect of KRPBE on Aβ-induced mitochondrial dysfunction (Fig. 7). Similar to this study, treatment with PYC protected mitochondrial membrane potential by reducing oxidative stress in Wister rats with spinal cord injury. These results ultimately inhibited neuronal cell death by inhibiting the activation of the mitochondrial apoptotic pathway and contributed to a significant recovery of the motor function of the hind limbs in rats [63]. PCA restored mitochondrial membrane potential in human umbilical vein endothelial cells (HUVECs) compared to palmitic acid-treated cells [64]. Therefore, treatment with KRPBE is thought to have a protective and restorative effect on mitochondrial function and inhibits neuronal cell death by protecting mitochondrial membrane.

When microglia are activated by endogenous pro-inflammatory mediators, such as TNF-α, IL-1β, Aβ peptides and tau oligomers, they release neurotoxic substances such as nitric oxide (NO), ROS, reactive nitrogen species (RNS), and cytokines [65]. TNF-α and IL-1β are significantly increased in AD patients and have harmful effects on memory and cognition inducing tau protein phosphorylation and the formation of NFTs [66]. In addition, c-Jun N-terminal kinase (JNK) is induced by TNF-α and Aβ peptides, and p-JNK promotes phosphorylation of serine residues of insulin receptor substrate 1 (IRS-1) (p-IRS-1Sser), which negatively affects downstream effectors such as the PI3K/Akt cascade [67-69]. In addition, TNF-α activates IκB kinase (IKK), which phosphorylates IκB and results in the activation of the nuclear factor kappa-light-chain-enhancer of activated B cell (NF-κB) signaling pathway, leading to the secretion of pro-inflammatory cytokines and subsequent inflammation [70]. Therefore, the protective effect of KRPBE against Aβ-induced neuroinflammation was investigated (Fig. 8). Administration of KRPBE was shown to decrease the expression level of some neuroinflammatory factors induced by AB and tau hyperphosphorylation. Treatment of PYC attenuated the expression of NF-κB, IL-1β, TNF-α, cyclooxygenase-2 (COX-2) and inducible nitric oxide synthase (iNOS), contributing to its neuroprotective effect in a 1-methyl-4-phenyl-1,2,3,6-tetrahydro pyridine (MPTP)-induced Parkinson’s disease (PD) model [71]. In addition, pine bark extract (PBE) containing large amounts of phenolic compounds, flavonoids, and proanthocyanidins significantly inhibited the activation of microglia and decreased the expression level of pro-inflammatory cytokines [72]. Therefore, the treatment of KRPBE is considered to have an improvement effect on cognitive function by regulating the expression level of Aβ-induced neuroinflammatory factors.

Aβ activates the pro-apoptotic pathway in AD [54]. Overexpression of BCl-2 is a major inhibitor of apoptosis because it prevents apoptosis by blocking the translocation of cytochrome C from the mitochondria to the cytoplasm. On the other hand, overexpression of BAX increases apoptosis by signaling apoptosis [73, 74]. It also induces the activation of caspase-3 and caspase-9 which are involved in the caspase cascade [45, 54]. Therefore, we investigated the anti-apoptotic effect of KRPBE extract and found that it suppressed the apoptotic protein expression levels and reduced the expression level of the BAX/BCl-2 ratio (Fig. 9). *P. densiflora* bark inhibited apoptosis by downregulating mRNA expression levels of caspase 3 and BAX in SD rat pups with selenite-induced cataracts. In addition, the apoptosis mechanism was improved by maintaining the mRNA expression level of BCl-2, an anti-apoptotic factor, at an almost normal level [49]. Taxifolin, a major bioactive component of KRPBE, ultimately reversed neurobehavioral disorders through hepatic and neuroprotective roles by regulating antioxidant capacity, pro-inflammatory mediators and levels of BAX and BCl-2 against thioacetamide-induced hepatic encephalopathy [75]. In addition, PCA showed neuroprotective properties by inhibiting the activation of neuro-oxidative damage and inflammatory response induced by arsenic exposure, downregulating BAX and caspase 3, and upregulating BCl-2 and BDNF [76]. Therefore, KRPBE contributes to the neuroprotective effect by suppressing the death of brain neurons by reducing the expression level of the BAX/BCl-2 ratio.

BDNF is mediator that plays a crucial role in neuronal differentiation, maturation and survival, and synaptic plasticity. Furthermore, BDNF binds to the tropomyosin receptor kinase B (TrkB) and activates several intracellular proteins, including ERK [77]. Activated proteins inhibit pro-apoptotic protein and activate CREB transcription factor [34]. CREB regulates the transcription of BDNF, in particular BDNF transcript IV, and plays an important role in learning and memory progress [78]. In addition, oligomeric Aβ_1-42_ significantly downregulates BDNF transcripts IV and V, reducing the pathology of AD in human cortical tissue [79]. These processes lead to neurological dysfunction and play a crucial role in the pathogenesis of AD [80]. Therefore, the regulatory effect of KRPBE was investigated through the CREB/BDNF pathway (Fig. 10). KRPBE treatment significantly increased the expression levels of p-CREB and BDNF compared to the Aβ induction group. Similar to these results, treatment with ethanolic pine needle extract increased the expression levels of BDNF and p-CREB against scopolamine-induced hippocampal dysfunction [81]. In addition, PCA, a physiologically active substance of KRPBE, showed an anti-apoptotic effect by significantly increasing the BDNF level, the CREB target gene, under chronic intermittent hypoxia [82]. Based on these results, the administration of KRPBE significantly increased the antioxidant system capacity and mitochondrial activity in Aβ-induced cognitive decline mice. KRPBE also significantly reduced Aβ-induced neuroinflammation and apoptosis-related factors. Furthermore, the treatment with KRPBE considerably improved cognitive deficits by upregulating the BDNF/CREB pathway, which contributes to neuronal survival and synaptic plasticity.
